# PROS1 is a crucial gene in the macrophage efferocytosis of diabetic foot ulcers: a concerted analytical approach through the prisms of computer analysis

**DOI:** 10.18632/aging.205732

**Published:** 2024-04-10

**Authors:** Hongshuo Shi, Zhicheng Zhang, Xin Yuan, Guobin Liu, Weijing Fan, Wenbo Wang

**Affiliations:** 1 Department of Peripheral Vascular Surgery, Institute of Surgery of Traditional Chinese Medicine, Shuguang Hospital Affiliated to Shanghai University of Traditional Chinese Medicine, Shanghai, China; 2 Dongying People’s Hospital (Dongying Hospital of Shandong Provincial Hospital Group), Dongying, Shandong, China; 3 The Second Affiliated Hospital of Shandong University of Traditional Chinese Medicine, Jinan, Shandong, China

**Keywords:** bioinformatics, machine learning, macrophage efferocytosis, diabetic foot ulcer, PROS1

## Abstract

Background: Diabetic foot ulcers (DFUs) pose a serious long-term threat because of elevated mortality and disability risks. Research on its biomarkers is still, however, very limited. In this paper, we have effectively identified biomarkers linked with macrophage excretion in diabetic foot ulcers through the application of bioinformatics and machine learning methodologies. These findings were subsequently validated using external datasets and animal experiments. Such discoveries are anticipated to offer novel insights and approaches for the early diagnosis and treatment of DFU.

Methods: In this work, we used the Gene Expression Omnibus (GEO) database’s datasets GSE68183 and GSE80178 as the training dataset to build a gene model using machine learning methods. After that, we used the training and validation sets to validate the model (GSE134431). On the model genes, we performed enrichment analysis using both gene set variant analysis (GSVA) and gene set enrichment analysis (GSEA). Additionally, the model genes were subjected to immunological association and immune function analyses.

Results: In this study, PROS1 was identified as a potential key target associated with macrophage efflux in DFU by machine learning and bioinformatics approaches. Subsequently, the key biomarker status of PROS1 in DFU was also confirmed by external datasets. In addition, PROS1 also plays a key role in macrophage exudation in DFU. This gene may be associated with macrophage M1, CD4 memory T cells, naïve B cells, and macrophage M2, and affects IL-17, Rap1, hedgehog, and JAK-STAT signaling pathways.

Conclusions: PROS1 was identified and validated as a biomarker for DFU. This finding has the potential to provide a target for macrophage clearance of DFU.

## INTRODUCTION

Diabetic foot ulcers (DFUs) represent one of the most devastating complications of diabetes, posing a significant challenge in healthcare. Patients frequently endure ongoing pain, infection, and lengthy rehabilitation, thereby imposing substantial financial and physical burdens. Beyond the medical expenses entailed in treatment, DFUs can diminish work capacity, lower quality of life, and give rise to mental health concerns, thereby subjecting patients and their families to considerable psychological stress [1]. Frequent exacerbation of DFU is often attributed to extensive changes associated with diabetes, including neuropathy and vascular issues [2]. The prevalence of diabetic foot ulcers (DFUs) ranges from approximately 15% to 25% throughout the lifespan of a diabetic patient. Notably, the incidence of DFUs increases from 19% to 34% if the patient has a history of foot injury or infection. Alarmingly, amputation may eventually be necessary in about 17% of these cases [3]. In 2019, direct global healthcare expenditures related to diabetes amounted to approximately $700 billion. It is estimated that these costs are projected to rise to $825 billion by 2030, with healthcare costs associated with diabetic foot problems accounting for one-third of total diabetes management spending [4]. Diabetic foot ulcers (DFUs) have a very complicated etiology that is typically brought on by peripheral vascular diseases and distal neuropathy that affect the lower limbs. Treatment choices are made even more complex and difficult by the concurrent infection that occurs in over half of DFU cases [4]. Therefore, in order to improve the outcome and prognosis for patients with DFU, it is imperative to study novel DFU biomarkers and avoid the creation of chronic wounds [4].

One of the main cells that control the healing process of wounds is the macrophage [5]. Without macrophages, wounds will not heal as quickly, blood vessel creation will be hindered, collagen deposition will be lowered, and cell proliferation will be slowed down [5]. It has been demonstrated that trauma-induced macrophages can change during the phagocytosis of apoptotic cells from a pro-inflammatory M1 phenotype to an anti-inflammatory M2 phenotype, contributing to the elimination of apoptotic cells [5]. Excessive blood glucose levels can cause high levels of advanced glycosylation end-products (AGEs) being produced, which are formed when proteins or lipids are exposed to sugar and undergo glycosylation [6]. AGEs and macrophages expressing high levels of the receptor for AGE (RAGE) accumulate in the diabetic wound environment. Research conducted *in vitro* has demonstrated that AGEs decrease the phagocytic ability of M1 macrophages. One of the main mechanisms causing macrophages to change into M2 phenotypes is efferocytosis of phagocytosed apoptotic cells [7]. Better wound healing is achieved when anti-RAGE antibodies are administered *in vivo* to wounds, as this increases phagocytic activity (efferocytosis) and encourages macrophage transition to the M2 phenotype. This shows that AGE concentrations in wounds can prevent phenotypic change and expedite healing [8]. The intracellular macrophages extracted from diabetic mouse wounds exhibit impaired efferocytosis function, causing an increase in apoptotic cells in the wound [9, 10]. Moreover, the efferocytosis of apoptotic cells drives macrophages towards the M2 phenotype, which could be the main driving factor for wound healing transition [11]. However, currently there is a lack of relevant research on the key targets of macrophage efferocytosis in DFU.

Precision medicine is swiftly evolving into an approach that customizes medical care for specific small groups or even individual patients, considering their genetic, environmental, and habit factors. This approach heavily depends on advancements in systems biology and omics disciplines [12]. The key is to understand and treat diseases by integrating multimodal or multi-omics data from individuals, in order to make informed decisions tailored to each patient. Meanwhile, the rapid development of computer science has led to the emergence of technologies capable of storing, processing, and analyzing these complex datasets. *In vivo* administration of anti-RAGE antibodies to wounds enhances phagocytic activity (efferocytosis) and promotes the shift of macrophages to the M2 phenotype, thereby promoting better wound healing. This suggests that high levels of AGEs in wounds impede phenotypic shift and timely repair [13].

Through the use of clinical annotations and high-throughput transcriptome sequencing data, the Diabetic Foot Unit (DFU) program enables us to investigate alterations in transcriptional patterns and related molecular pathways linked to DFU in biological research [14]. Numerous researches have examined molecular markers linked to the development of DFU using gene expression data taken from the Gene Expression Omnibus (GEO) database [15, 16]. Advancements in high-throughput sequencing technologies and the incorporation of machine learning in medicine have led to the emergence of novel methodologies for investigating molecular targets across various diseases [14, 17]. Through the application of bioinformatics technology, genes related to DFU that are associated with macrophage efferocytosis have been identified. In order to accurately identify the biomarkers associated with DFU, we utilized machine learning algorithms and screened key gene models.

**Figure 1:**
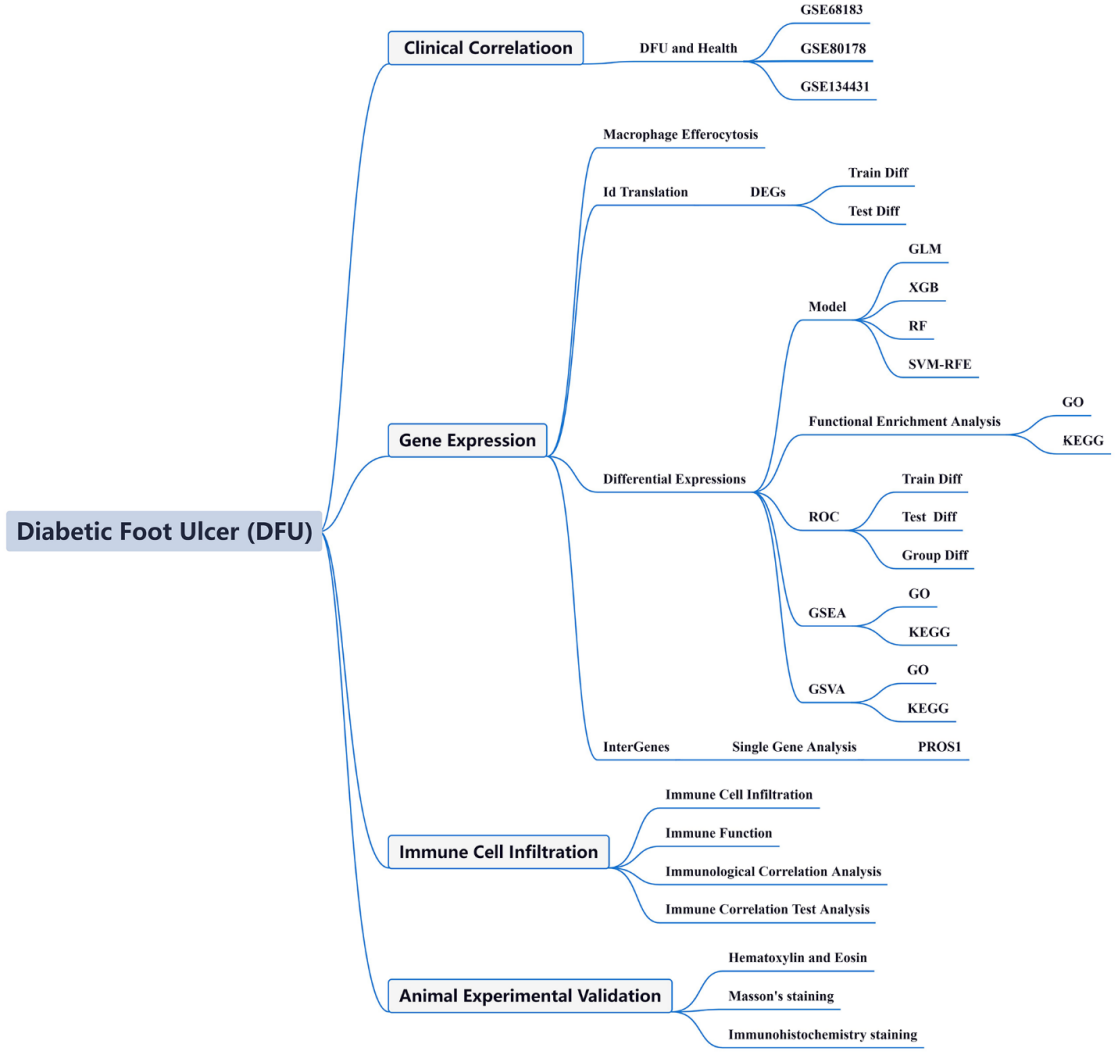
Flow chart of this study.

Following this, candidate genes displaying strong correlations with immune infiltration were validated using a separate, independent validation dataset. Subsequently, animal experiments were conducted to affirm the crucial genes identified. Figure 1 depicts the study’s flowchart.

## MATERIALS AND METHODS

### Raw data

The GEO database provided the microarray data for the mRNA expression profiles associated with DFU. This work made use of the GEO datasets GSE68183, GSE80178, and GSE134431 as well as the matching platform files GPL16686 and GPL18573. The training datasets in this study were GSE68183 and GSE80178, while the validation set was GSE134431. Three DFU patient samples and three control samples were included in GSE68183. Three samples of normal skin and nine samples from DF patients are included in GSE80178 [18]. The GSE134431 dataset comprised ulcer samples from 13 patients with diabetic foot (DF) and skin samples from 8 DF patients [19]. Comprehensive details regarding the mentioned datasets are provided in Table 1. The associated genes of macrophage efferocytosis (MERGs) were obtained from the GeneCards database (Supplementary Table 1).

**Table 1:** Dataset information.

**Dataset**	**Platform**	**Count**	**DFU**	**Control**
GSE68183	GPL16686	6	3	3
GSE80178	GPL16686	12	9	3
GSE134431	GPL18573	21	13	8

### Data filtering and processing

By using the probe annotation file, the downloaded probe matrix was transformed into a gene expression matrix. When a gene corresponded to more than one probe, the gene’s final expression level was determined by averaging these probes [20]. The batch effect between the GSE68183 and GSE80178 datasets was reduced using the SVA software after the datasets had been normalized. Principal component analysis (PCA) was then employed to evaluate batch effect removal.

### Analysis of differentially expressed genes (DEGs)

We aligned the MERGs and conducted a comparative analysis on them. We identified pertinent differential MERGs, and furthermore, we conducted a differential analysis based on the expression of core genes.

### Construction and validation of prediction models using various machine learning methods

We used the “caret” R package to build machine learning models based on two different MERGs clusters, including Support Vector Machine (SVM) [21], Extreme Gradient Boosting (XGB) [22], Generalized Linear Model (GLM) [23], and Random Forest (RF) [24]. Receiver operating characteristic (ROC) curves assess the diagnostic utility of these biomarkers. The “total score” represents the cumulative score of the predictor variables. In addition, we utilized calibration curves and decision curve analysis (DCA) to assess the predictive power of the histogram model. In addition, we performed variance analysis and ROC validation for each gene in the model, utilizing both the training and validation sets.

A generalized linear model (GLM) is an extension of a linear model that better accommodates different types of data distributions by using a link function to establish a relationship between the expected value of the response variable and a linear combination of the predictor variables. XGBoost (Extreme Gradient Boosting) is a machine learning library based on the gradient boosting decision tree algorithm, known for its efficient implementation. XGBoost can be used to solve a variety of tasks such as classification, regression, and sorting, and has achieved notable success in several machine learning competitions. Support Vector Machines (SVMs) are powerful supervised learning models that are primarily used to solve classification and regression problems. By transforming the data into an optimization problem and finding the optimal separating hyperplane on the training data, SVMs are able to perform pattern recognition and regression analysis efficiently. Random Forest (RF) is an integrated learning algorithm that improves the accuracy and robustness of classification or regression by constructing multiple decision trees and combining their outputs. Due to its excellent performance and ease of use, Random Forest has become one of the preferred algorithms in many machine learning practices.

### Pathway and functional enrichment analysis

The R package clusterProfiler was utilized to do enrichment analysis for the Kyoto Encyclopedia of Genes and Genomes (KEGG) [25] and Gene Ontology (GO) [25]. Moreover, to identify possible pathways, Gene Set Enrichment Analysis (GSEA) was carried out [25]. Gene set variation analysis (GSVA) is a non-parametric, unsupervised analytical method mostly used to assess gene set enrichment in sequencing data [25].

### Immunocyte infiltration analysis

The CIBERSORT method and the immune cell LM22 gene set were utilized to determine the relative abundance of 22 different lymphocyte subtypes in both the normal sample and each diabetic foot patient [26].

### Correlation analysis between diagnostic biomarkers and immune cells

We performed a Spearman correlation analysis with the aim of exploring in depth the association between diagnostic biomarkers and infiltrating immune cells. This analysis can help us better understand the role of biomarkers in immune cell infiltration, thus providing more comprehensive information and guidance for disease diagnosis and treatment [27].

### Data availability

All data generated have been incorporated into the published manuscript. The gene expression datasets produced during this investigation are accessible in the NCBI Gene Expression Omnibus (GEO) database under the accession numbers: GSE68183, GSE80178, and GSE134431. For additional information, please reach out to the authors at Hongshuo Shi’s email address: jf17510413109@163.com.

## RESULTS

### GEO data processing

**Figure 2:**
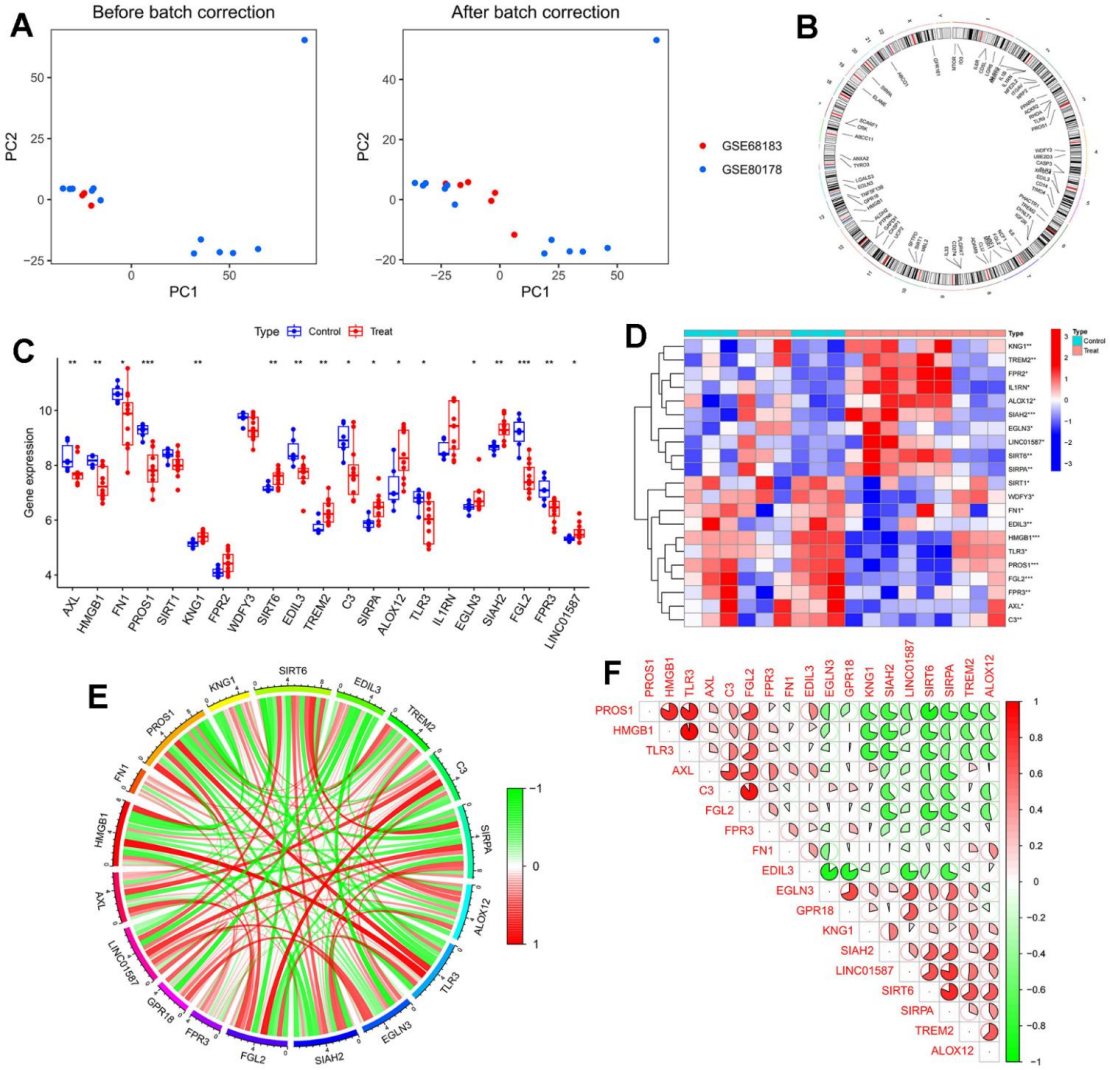
**Dataset preprocessing and difference analysis.** (**A**) Principal component analysis (PCA) analysis before batch effect removal; (**B**) Heatmap of deGlnMRG; (**C**) The location of MERGs on chromosomes; (**D**) The expression levels of MERGs; (**E**) Gene relationship network diagram of deMERGs; (**F**) Correlation analysis of deMERGs. Red and green colors represent positive and negative correlations, respectively. The correlation coefficient was expressed as the area of the pie chart.

In this paper, we merged two DFU datasets, GSE68183 and GSE80178, and the datasets included a total of 6 normal skin samples and 12 DFU samples. In Figure 2A, we show the gene expression level assessment and PCA results of each sample before and after removing the batch effect. In Figure 2B, we calculated the chromosomal locations of MERGs and indicated them with circles. We identified 21 differentially expressed MERGs (deMERGs). Among these MERGs, KNG1, TREM2, FPR2, IL1RN, ALOX12, SIAH2, EGLN3, LINC01587, SIRT6, and SIRPA were expressed at higher levels in DFU. In contrast, SIRT1, WDFY3, FN1, EDIL3, HMGB1, TLR3, PROS1, FGL2, FPR3, AXL, and C3 were expressed at significantly lower levels in DFUs than in normal controls (see Figure 2C, 2D). Subsequently, correlation analysis of these genes was performed in this paper (see Figure 2D, 2E).

### Building and evaluating machine learning models

**Figure 3:**
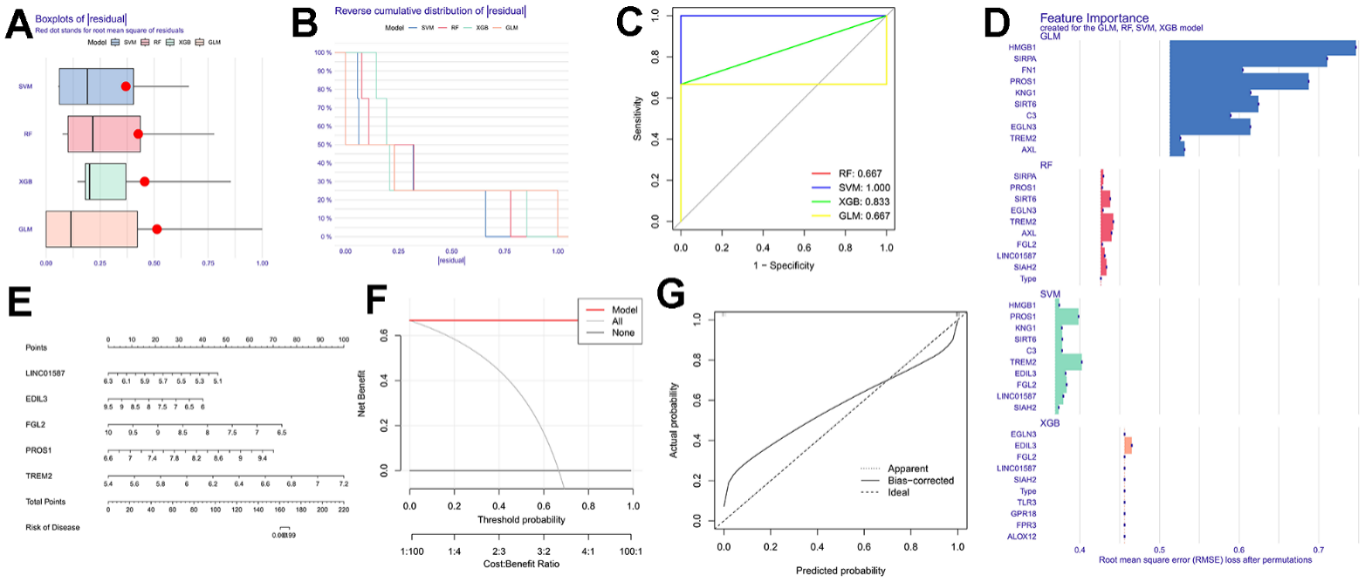
**Construction of machine learning models.** (**A**) The cumulative residual distribution of the four models; (**B**) Residual boxplots of the four machine learning models, where the red dots indicate the root mean square of the residuals; (**C**) ROC analysis of four machine learning models with 5-fold cross-validation in the test set; (**D**) The important features in SVM, RF, XGB, and GLM; (**E**) Construction of a nomogram to predict DFU risk based on a 5-gene SVM model; (**F**, **G**) Calibration curves.

In this study, a machine learning model was constructed utilizing deMERGs (refer to Figure 3A). Residual distribution analysis indicated that SVM exhibited the highest residuals among the four models (see Figure 3B). ROC analysis demonstrated that SVM achieved an AUC value of 1.000, outperforming the other machine learning models (see Figure 3C). Figure 3D presented the top 10 significant feature variables for each model based on root mean square error. To assess the predictive performance of SVM models (LINC01587, EDIL3, FGL2, PROS1, and TREM2), line plots were plotted (see Figure 3E). Detailed model information can be found in Table 2. Prediction accuracy of the line plot models was evaluated using calibration curves and decision curve analysis (DCA). Calibration curves illustrated the minimum error between actual DFU cluster risk and predicted risk (see Figure 3F). Decision curve analysis demonstrated high accuracy of the line graphs, making them valuable for clinical decision-making (see Figure 3G).

**Table 2:** Model information.

**Variable**	**Permutation**	**Dropout_loss**	**Label**
LINC01587	0	0.379102009	SVM
EDIL3	0	0.382035832	SVM
FGL2	0	0.383280431	SVM
PROS1	0	0.398267204	SVM
TREM2	0	0.402331623	SVM

### Differential validation of model genes

**Figure 4:**
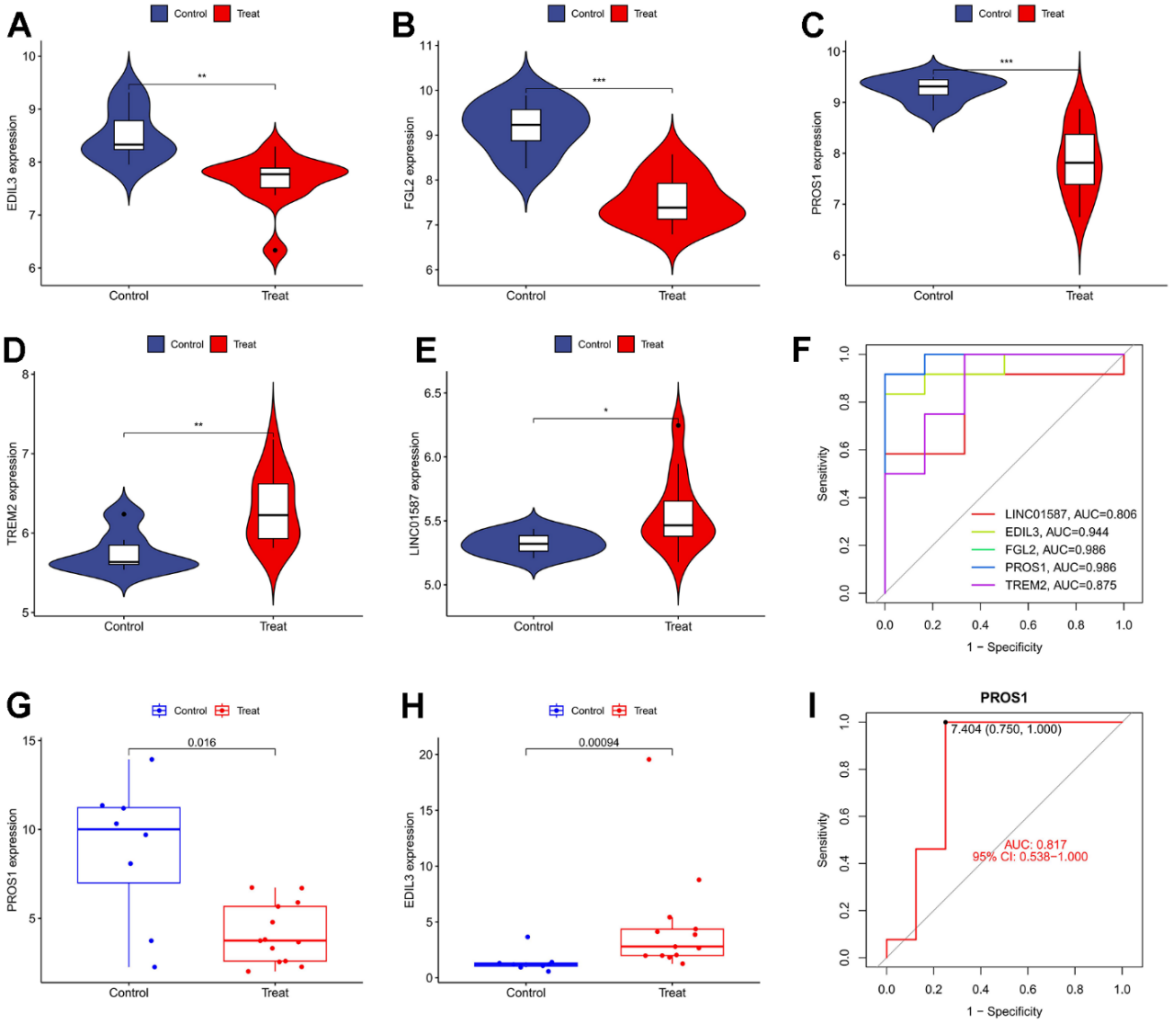
**The analysis of genetic differences and diagnostic efficacy of model genes.** (**A**) Differential expression of EDIL3; (**B**) Differential expression of FGL2; (**C**) Differential expression of PROS1; (**D**) Differential expression of TREM2; (**E**) Differential expression of LINC01587; (**F**) The ROC curve of SVM model; (**G**) Validating the differential expression of PROS1 in the experimental group; (**H**) Validating the differential expression of EDIL3 in the experimental group; (**I**) The ROC curve of PROS1 in the validation group.

In the training set, we observed higher expression levels of EDIL3 (Figure 4A), FGL2 (Figure 4B), and PROS1 (Figure 4C) in the normal group, whereas TREM2 (Figure 4D) and LINC01587 (Figure 4E) showed higher expression levels in the DFU group. The ROC curves for these genes are depicted in Figure 4F. In the variance analysis of the validation set, we found that PROS1 (Figure 4G) expression was down-regulated in DFU, consistent with the results from the training set, while the expression pattern of EDIL3 (Figure 4H) was opposite to that observed in the training set. Consequently, PROS1 may serve as a key marker in DFU. The ROC curves of PROS1 in the validation set also demonstrated its strong diagnostic ability (Figure 4I).

### Analysis of DEGs associated with PROS1 and enrichment analysis

**Figure 5:**
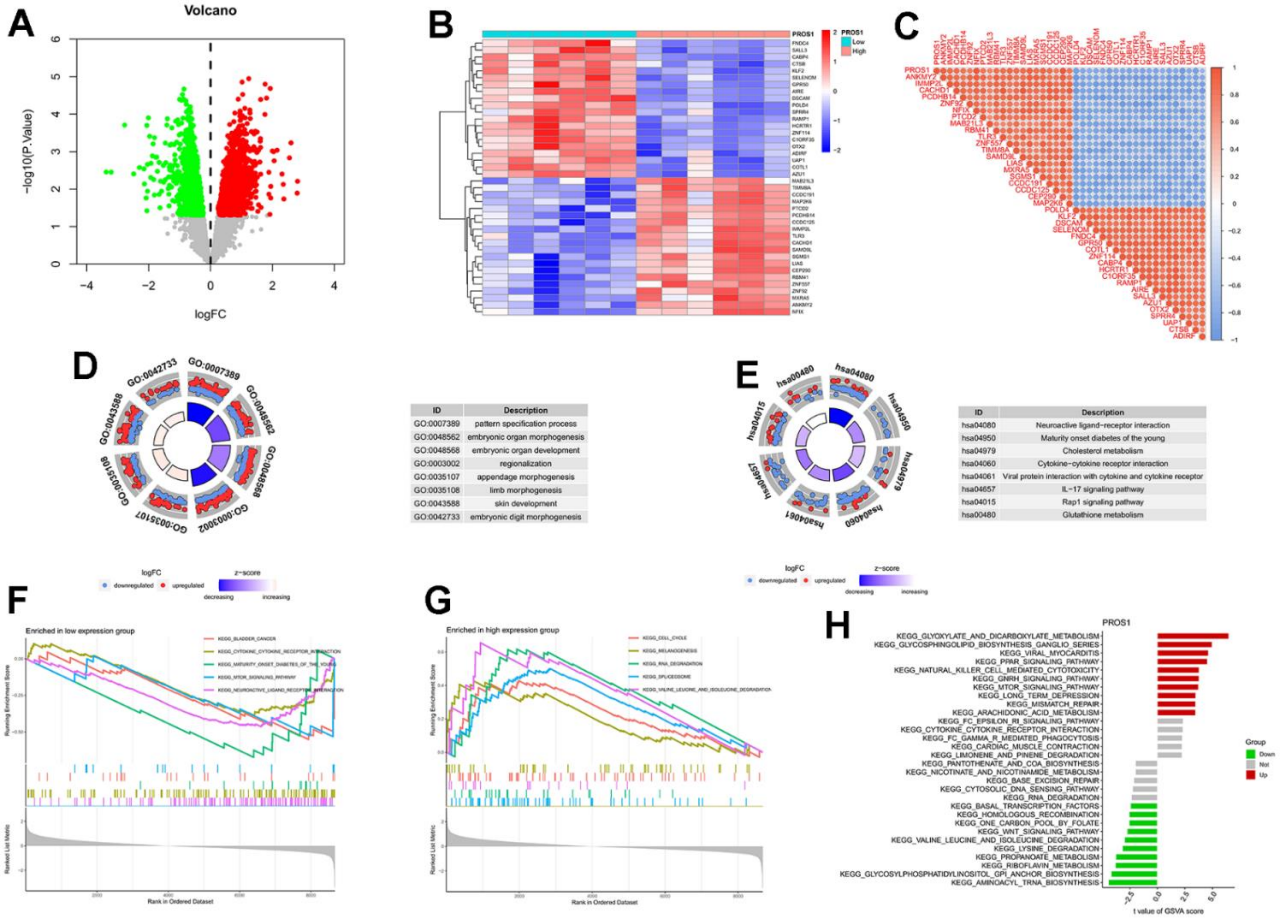
**Differential analysis and enrichment analysis of genes related to PROS1.** (**A**) Volcano plot for differential analysis. (**B**) Differential analysis heatmap; (**C**) Correlation analysis heatmap; (**D**) Differential gene GO analysis; (**E**) Differential gene KEGG analysis; (**F**) GSEA analysis of downregulated genes; (**G**) GSEA analysis of upregulated genes; (**H**) PROS1-related GSVA analysis.

Based on the expression levels of PROS1, we conducted a differential analysis of genes in the training set. The results of this analysis were visualized through volcano plots (Figure 5A), heat maps (Figure 5B), and correlation heat maps (Figure 5C). Enrichment analysis revealed genetic biological processes involving pattern specification (GO: 0007389), embryonic organ morphogenesis (GO: 0048562), and embryonic organ development (GO: 0048568) (Figure 5D). KEGG enrichment analysis indicated that differentially expressed genes were implicated in pathways such as the IL-17 signaling pathway (hsa04657), Rap1 signaling pathway (hsa04015), and hedgehog signaling pathway (Figure 5E), suggesting a close association between PROS1 and inflammation. GSEA analysis demonstrated that the cell cycle, melanogenesis, and RNA degradation pathways were activated in the PROS1 high-expression group (Figure 5F). Conversely, in the PROS1 low-expression group, pathways related to bladder cancer, cytokine-cytokine receptor interactions, and youth maturity-onset diabetes were active (Figure 5G). GSVA analysis further indicated that PROS1 was closely linked to glyoxylate and dicarboxylic acid metabolism, as well as aminoacyl tRNA biosynthesis (Figure 5H).

### Analysis of immune function and immune cell infiltration

**Figure 6:**
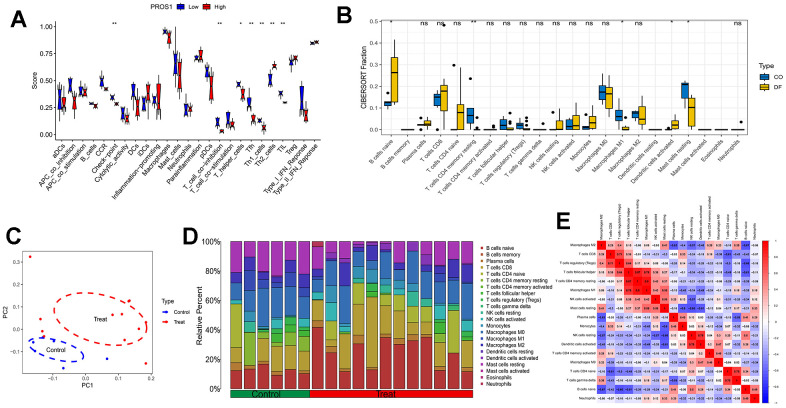
**Immune analysis.** (**A**) Immune functional analysis of the PROS1 gene; (**B**) DFU immune infiltration analysis; (**C**) PCA analysis of immune infiltration results; (**D**) Immune cell distribution; (**E**) Immune cell correlation analysis.

Immunofunctional analyses revealed that PROS1 may be associated with immune checkpoints, T-cell synergistic suppression, T-helper cells, Tfh, Th1 cells, Th2 cells, and tumor-infiltrating lymphocytes (TILs) (Figure 6A). Compared to controls, DFU samples exhibited an increase in B-cell naïve and dendritic cell-activated phenotypes, along with a decrease in the number of T-cells CD4 memory-resting, regulatory T-cells (Tregs), follicular helper T-cells, macrophage M1, and resting mast cells (Figure 6B). The distribution of immune cells and the results of principal component analysis (PCA) are illustrated in Figure 6C, while the distribution of immune cells is demonstrated in Figure 6D. Further analysis of the relationship between the 22 immune cells in all samples revealed a significant positive correlation between macrophage M1 and T-cell CD4 memory resting phenotypes, whereas B-cell naïve phenotypes exhibited a significant negative correlation with macrophage M2 (Figure 6E).

**Figure 7:**
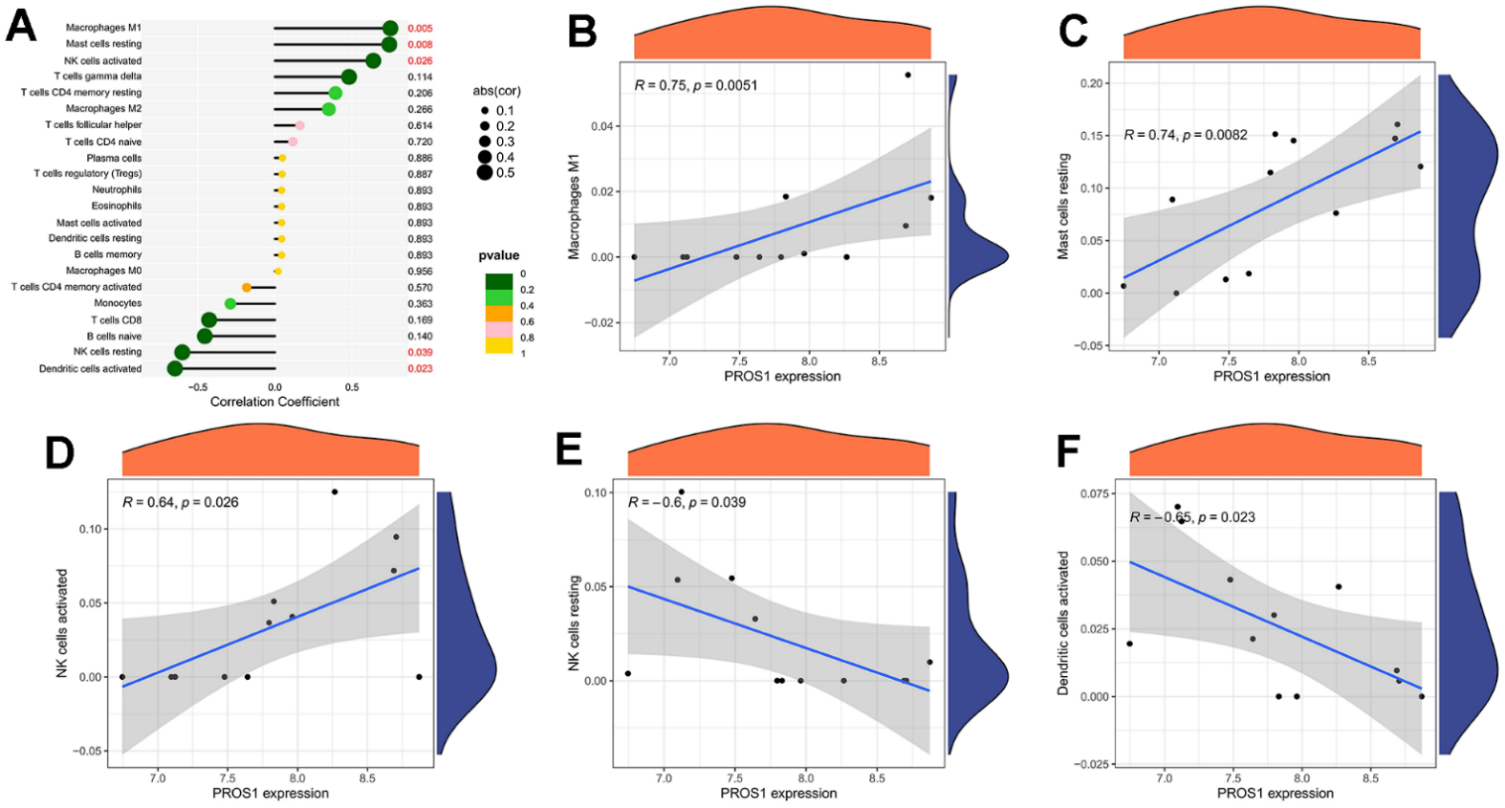
**Immune cell correlation analysis of PROS1.** (**A**) Correlation analysis; (**B**) PROS1 correlation analysis with Macrophages M1; (**C**) PROS1 correlation analysis with mast cells resting; (**D**) PROS1 correlation analysis with NK cells activated; (**E**) PROS1 correlation analysis with NK cells resting; (**F**) PROS1 correlation analysis with dendritic cells activated.

The correlation analysis between the gene PROS1 and immune cells is depicted in Figure 7A. We observed significant positive correlations between PROS1 and macrophage M1 (r = 0.74, p = 0.005) (Figure 7B), resting mast cells (r = 0.75, p = 0.008) (Figure 7C), and activated NK cells (r = 0.64, p = 0.03) (Figure 7D). Additionally, PROS1 exhibited a negative correlation with resting NK cells (r = -0.65, p = 0.02) (Figure 7E) and activated dendritic cells (r = -0.60, p = 0.04) (Figure 7F).

## DISCUSSION

DFU stands out as a prevalent complication of diabetes, imposing significant burdens on patients while also presenting challenges to overall health, nursing protocols, and the social fabric [28]. The role of macrophage efferocytosis in DFU has also received extensive attention from researchers [28]. Leveraging machine learning and bioinformatics methodologies, the current study aimed to pinpoint PROS1 as a potential crucial target for DFUs from the standpoint of macrophage phagocytosis. The reliability of these findings is bolstered by the diminished expression of PROS1 observed in the validation set of DFU patients and in animal experimentation.

The difficulties in healing diabetic wounds can be attributed to complex factors, such as impaired blood vessel formation, abnormal inflammatory response, and dysfunctional macrophage efferocytosis [28]. Diabetes patients experience altered macrophage function, leading to a reduced ability to clear infections. Furthermore, their functionality during the later stages of repair is also affected, resulting in delayed healing processes [28]. In a mouse model mimicking diabetes, impaired phagocytosis of apoptotic cells resulted in the accumulation of apoptotic cells in wounds and maintained an inflammatory microenvironment [9]. Moreover, research indicates that efferocytosis effectively facilitates the transition of pro-inflammatory M1 macrophages to reparative M2 macrophages [29]. The transformation of macrophages plays a crucial role in inducing the efferocytosis function. Diabetic wounds produce various inflammatory factors and chemotactic factors in the wound microenvironment, such as AGE, MCP-1, DAMPs, and IL-1β, which mutually induce the NLRP3 and IL-1R1 signaling pathways. These events impede macrophage polarization and directly affect the process of the efferocytosis function [30, 31]. This study identified and validated that PROS1 may be a crucial gene influencing the macrophage efferocytosis phenotype in DFU.

PROS1 is located on chromosome 3q11.1 and is a vitamin K-dependent plasma protein that activates protein C to activate coagulation factors V and VIII while promoting the clearance of early apoptotic cells [32]. The members of the Tyro3/Axl/Mer (TAM) receptor tyrosine kinase (RTK) family serve as vital regulators of the innate immune system, playing a pivotal role in the efficient clearance of apoptotic materials during normal homeostatic processes [33, 34]. Gas6 and Pros1, the extensively researched TAM ligands, act as intermediary molecules, connecting phosphatidylserine (PtdSer) exposed on the surface of apoptotic cells with TAM receptors on macrophages. This interaction subsequently initiates phagocytosis [35]. PROS1 also serves a significant function in the process of blood clotting. The components of the hemostatic and fibrinolytic systems play an essential role in the wound healing process. In addition to their direct contribution to the formation of a clot, which acts as a barrier against bleeding and pathogens, their interaction with inflammatory cells also lays the foundation for antimicrobial activity, extracellular matrix degradation, migration and proliferation of keratinocytes, and wound contraction [36]. TAM receptors play a crucial role as key regulators of inflammatory responses. PROS1 functions as a ligand for TAM receptors. When PROS1 is deficient, there is an increase in the expression of pro-inflammatory cytokines such as TNF-α and CCL3 [37]. The expression of PROS1 shows a positive correlation with neutrophil count, activity, and oxidative burst, suggesting its potential as a therapeutic target for conditions like decompensated liver cirrhosis and sepsis [38]. PROS1 emerges as a promising targeted drug candidate for the treatment of inflammatory disorders, including spinal cord injury and ankylosing spondylitis [38]. In this study, PROS1 was found to be downregulated in the group with DFU, suggesting its potential as a protective factor against DFU. However, the specific protective role of PROS1 in DFU remains unclear and requires further investigation.

Numerous research studies have suggested potential associations between PROS1 and signaling pathways such as IL-17, Rap1, hedgehog, and JAK-STAT. The wound healing process is intricately regulated by a complex network of cytokines and growth factors that govern intercellular interactions. Specifically, inflammatory signals play a pivotal role in dictating various aspects of tissue repair and regeneration. Dysregulation of these signals often results in abnormal inflammatory responses, leading to the failure of re-epithelialization. These factors are crucial in the development of slow or non-healing skin ulcers, which are increasingly recognized as a significant global health concern [39]. Bioinformatics analysis has revealed that interleukin 17A (IL-17A) is prominently upregulated in wound tissues, primarily synthesized by Th17 cells. Research indicates that IL-17A plays a critical role in promoting wound epithelialization [40]. Rap1, belonging to the Ras family of small GTPases, is a highly conserved cytoplasmic protein. It governs signaling pathways crucial for cellular cytoskeleton organization and cell-cell adhesion [41, 42]. Rap1 is mobilized to the wound perimeters and tricellular junctions where it becomes activated. Researchers found that heightened Rap1 activity leads to enhanced recruitment of myosin to the wound edges, thus expediting the process of wound healing [43]. The Hedgehog pathway is essential for precise morphogenesis and embryonic development [44]. Recent studies have indicated that the use of Hedgehog pathway agonists can alleviate impaired angiogenesis in diabetic mice. In addition, metformin downregulates autophagy through the Hedgehog signaling pathway, thereby alleviating high glucose-induced endothelial dysfunction [45]. Research has shown that the Hedgehog signaling pathway promotes endothelial cell proliferation and accelerates skin wound healing [46]. Cytokines and growth factors play a crucial role in the wound healing process by initiating various signaling pathways, including JAK/STAT [47]. Up-regulation of the JAK/STAT pathway is essential in chronic wounds, particularly when its normal function is compromised, especially in environments characterized by cellular aging and reduced growth factor/receptor efficacy. When epidermal growth factor binds to its receptor, it triggers dimerization and tyrosine autophosphorylation of the receptor, thereby activating the JAK/STAT pathway. This mechanism fosters cell proliferation and migration, which are pivotal in wound healing [48].

Recently, the fusion of bioinformatics and machine learning has emerged as a crucial tool for identifying novel biomarkers associated with diabetic foot ulcers (DFUs) [49]. Prior research has hinted at MAPK3’s potential as a biomarker linked to tissue damage in DFUs [16]. However, investigations into macrophage secretion and its correlation with DFUs remain scarce. Our study leveraged machine learning and bioinformatics techniques to re-evaluate PROS1 and shed light on its associated functions and mechanisms. Validation sets and animal experiments both demonstrated a reduction in PROS1 expression within the DFU group, affirming the robustness of our findings. Despite these promising outcomes, it’s important to acknowledge certain limitations. Firstly, the clinical data were sourced from public databases, and some sample information was incomplete, notably the absence of clinicopathological characteristics in the GSE series. Secondly, while RNA sequencing data supported our bioinformatics analysis, future studies should validate these findings using clinical samples for enhanced reproducibility and generalizability. However, the acquisition of clinical samples posed challenges within our limited timeframe. Lastly, further exploration is warranted to unravel the precise mechanism of PROS1’s action in DFUs.

## CONCLUSIONS

The occurrence and progression of DFU result from intricate interactions among multiple factors, encompassing targets, immune cells, signaling pathways, and diverse biological processes. These regulatory mechanisms are collaborative and bidirectional in nature. In aggregate, this study has pinpointed PROS1 as a potentially significant regulator in DFU through the utilization of machine learning and bioinformatics methodologies. Subsequently, the pivotal role of PROS1 in macrophage efflux in DFU was confirmed through validation with external datasets, thus solidifying its significance as a biomarker.

## Supplementary Material

Supplementary Table 1
