# Supplementary material for: PROS1 is a crucial gene in the macrophage efferocytosis of diabetic foot ulcers: a concerted analytical approach through the prisms of computer analysis
Source: Aging (Albany NY). 2024 Apr 10;16(8):6883–97. doi: 10.18632/aging.205732 (PMC11087110; doi:10.18632/aging.205732)
Supplement: Supplementary Table 1 [file aging-16-205732-s001.pdf]

SUPPLEMENTARY TABLE

Supplementary Table 1. The related genes of macrophage efferocytosis.

MERTK	MEG8	NFE2L2	CD14	PLGRKT	GPR101	LOC124904141
TIMD4	LINC01151	TLR3	TGM2	MIR7-3HG	GPR18	LOC124904142
CD300LF	SMILR	ALDH2	S1PR5	C3	GAS5	LOC124904143
PLAUR	LINC01150	GAPDH	GABARAP	CASP1	MIRLET7C	LOC124904144
AXL	CERNA3	IL1RN	IL33	CASP3	MIRLET7D	LOC124904146
GAS6	ENSG00000255325	IL6R	DYNLT1	GATA2	LINC01587	LOC124907963
MIAT	PPARG	PTPN6	MIR126	ANXA2	MIR125A	LOC124907964
HAVCR1	CALR	CLU	ST2	SIRPA	MIR216A	LOC124907965
SMAD5-AS1	APOE	IL1B	MPO	ALOX12	MIR409	
HMGB1	NCF1	EGLN3	NLRP3	IFNB1	SNHG14	
RHOA	IGF2R	NTN1	ANXA1	XRCC4	MIR190B	
CD274	RMRP	LGALS3	FPR2	QPCTL	MIR379	
CD47	ITGB3	TNFSF13B	MSR1	XKR4	RAB4B-EGLN2	
PLG	ITGAV	CRK	WDFY3	MIR33A	MIR1293	
TYRO3	TREM2	IRF3	LINC02605	MIR33B	RNU2-1	
MIR34A	UCP2	NRF1	SIRT6	TMEM256-PLSCR3	PWAR4	
FN1	ACKR2	SFTPD	RAB17	PWAR1	TRA-TGC7-1	
PROS1	MBL2	UBE2D3	SERPINA1	LINC01672	MBL3P	
ITGB5	MIR21	SIAH2	PLCG1	PWAR6	TRA-TGC5-1	
SCARB1	NRP2	ALOX15B	VTN	SOD2-OT1	LOC124904135	
PHACTR1	TGFB1	LGR6	ABCG1	PGR-AS1	LOC124904136	
SIRT1	TGFB3	CD5L	ABCC11	TRE-TTC3-1	LOC124904137	
ABCA1	IL6	FGL2	EDIL3	LOC106694316	LOC124904138	
ADAM9	TLR9	FPR3	SCARF1	MTOR	LOC124904139	
KNG1	MFGE8	ID3	MIR148B	ELANE	LOC124904140	
